# Recent Advances in Anti-Mullerian Hormone (AMH)-Related Osteoporosis Research

**DOI:** 10.3390/biomedicines14020428

**Published:** 2026-02-13

**Authors:** Luojia Wang, Yuetong Guo, Rui Yan, Yan Yu, Heping Zhao, Yuzhu Yan

**Affiliations:** Clinical Laboratory of Honghui Hospital, Xi’an Jiaotong University, Xi’an 710054, China; luojiawang1288@163.com (L.W.); victoria2427839090@163.com (Y.G.); 13474546113@163.com (R.Y.); yu.yan74@163.com (Y.Y.); zhp64223@163.com (H.Z.)

**Keywords:** anti-Müllerian hormone (AMH), osteoporosis, bone metabolism, targeted therapy

## Abstract

Anti-Müllerian hormone (AMH), a member of the transforming growth factor-β (TGF-β) superfamily, has been widely recognized for its role in reproductive endocrinology and is regarded as one of the “gold standards” for evaluating ovarian age and fertility potential. In recent years, the focus of research on AMH has gradually expanded from the reproductive system to the skeletal system. Although the specific mechanism of its action in bone-metabolism-related diseases and associated signaling pathways still requires in-depth exploration, existing studies have confirmed—through cell experiments, animal models, and clinical data—the important role of AMH in maintaining bone health. Here, the significance of AMH in research on female osteoporosis is reviewed, the current signaling pathway mechanisms by which AMH regulates bone metabolism are summarized, and the relevant clinical research results are discussed. This work features three unique contributions: first, the logical progression of AMH research from reproductive regulation to bone metabolism is explicitly clarified; second, multi-level evidence is integrated to form a complete regulatory network, avoiding fragmented discussions of individual findings; and third, concrete clinical translation pathways and targeted solutions for existing limitations are proposed, rather than merely outlining general directions. This review aims to identify new biomarkers for the early screening of osteoporosis and therapeutic targets, ultimately promoting the formulation of personalized prevention and treatment strategies. Additionally, as a key factor linking ovarian function and bone health, the AMH research concepts and methods summarized herein can be extended to other hormone-related bone metabolism disorders.

## 1. Introduction

Osteoporosis, defined as a skeletal disorder characterized by compromised bone strength predisposing to an increased risk of fracture, is a major public health problem throughout the world [1]. Clinical diagnosis relies primarily on bone mineral density (BMD) measured via dual-energy X-ray absorptiometry (DXA). A BMD T-score ≤ −2.5 in individuals aged 50 and above is one of the key diagnostic indicators [2]. Globally, one-third of women and one-fifth of men over 50 years of age experience osteoporotic fractures during their lifetime [3]. In terms of disease hazards, hip fractures and clinical vertebral fractures are the most serious complications of osteoporosis, both directly associated with increased morbidity and mortality. Among these, hip fractures are the most severe, with a mortality rate of 24% within one year; they also cause reduced mobility and loss of independence in daily living, not only leading to pain and decreased activity ability but also significantly increasing the long-term care needs of patients [3,4]. Thus, osteoporotic fractures substantially reduce patients’ quality of life as well as imposing a heavy medical burden on public health systems.

Existing diagnostic methods have obvious limitations. The diagnosis of osteoporosis still depends on BMD detection, yet the decline in BMD often lags behind bone metabolic disorders. Approximately 70% of osteoporotic fractures occur in individuals whose BMD does not meet the diagnostic criteria for osteoporosis (T-score ≤ −2.5), so relying solely on BMD screening tends to miss high-risk patients [5]. Commonly used risk assessment tools (e.g., FRAX) fail to incorporate key factors such as fall history and detailed past fracture information, resulting in poor predictive accuracy in young postmenopausal women and male populations. Additionally, there is no unified standard for the target population and threshold setting of different assessment tools, which may lead to under-screening or over-screening of some high-risk groups [6]. In recent years, genomics research in the field of osteoporosis risk prediction has made significant strides [7,8]. Several large-scale international collaborative projects, such as the Genetic Factors for Osteoporosis Consortium (GEFOS), have expanded the number of SNP loci associated with BMD and fracture risk from hundreds to thousands through meta-analyses of hundreds of thousands or even millions of individuals [7]. These studies have not only confirmed numerous established bone metabolism pathways (such as the Wnt signaling pathway and the RANK-RANKL-OPG pathway) but also identified entirely novel candidate genes and biological pathways whose functions remain to be elucidated. Concurrently, polygenic risk scores (PRSs) represent the most promising translational avenue in recent osteoporosis genetic research. Studies have demonstrated that integrating PRSs into models incorporating BMD and clinical risk factors significantly enhances the accuracy of fracture risk prediction [9]. However, current genomics research pertaining to osteoporosis prediction continues to face challenges including low generalizability, a lack of longitudinal studies, and insufficient dynamic risk assessment [10]. In the field of treatment, due to gaps in clinical practice, even among women aged 65 years and above who are clearly recommended for screening, a large number still do not undergo BMD testing, and the proportion of patients receiving standardized treatment (e.g., drug intervention or rehabilitation management) promptly after fractures is also low [11,12]. Furthermore, the implementation of basic preventive measures (such as calcium and vitamin D supplementation or exercise intervention) is greatly affected by patient compliance, making it difficult to promote them widely and effectively in clinical practice [13]. Therefore, identifying new regulatory factors for early prediction of bone metabolic abnormalities and personalized treatment guidance has become a key priority in current osteoporosis research.

In women, bone mass gradually decreases during middle age, and particularly after menopause, the rate of BMD decline accelerates significantly, accompanied by accelerated bone turnover [14,15,16]. Since BMD remains relatively stable in premenopausal women, there is currently no reliable and effective method to screen for high-risk women who may develop osteoporosis or osteopenia in the future [17]. To achieve effective and efficient early intervention, two prerequisites must be met: (1) identifying women who are predisposed to substantial bone resorption and (2) determining whether bone resorption has already commenced or is imminent. Ovarian reserve refers to the number of remaining oocytes in the ovaries [18]. Given the confirmed role of ovarian function decline in the pathogenesis of postmenopausal osteoporosis, a number of ovarian function markers have been proven to be associated with female bone health, such as follicle-stimulating hormone (FSH), basal estradiol, and anti-Müllerian hormone (AMH) [19,20,21,22,23]. AMH is an ovary-specific growth factor secreted by growing small follicles and is independent of gonadotropins [24]. Serum AMH levels correlate closely with antral follicle count: they peak at 20 to 25 years, decline gradually with age, and become undetectable at menopause [25]. Therefore, unlike follicle-stimulating hormone, inhibin B, and estrogen levels, serum AMH levels remain relatively stable throughout the menstrual cycle [26]. The core advantages of AMH are its earliness and stability. First, AMH decreases much earlier than traditional indicators such as FSH and estrogen during reproductive aging. Since the menopausal transition (MT) is a critical period for accelerated bone resorption, AMH can provide an earlier warning of bone resorption risk, allowing more time for preventive intervention [27]. Second, AMH is secreted by small ovarian follicles and is independent of gonadotropins, so its levels remain relatively stable during the menstrual cycle. In contrast, FSH and estrogen fluctuate with the menstrual cycle, making AMH test results more reliable and reducing errors caused by differences in testing time [28]. With minimal diurnal and menstrual cycle fluctuations, AMH has no strict testing time requirements. Additionally, as it mainly functions in early follicular development, AMH is an earlier indicator of ovarian function than estrogen and can predict menopause and ovarian function decline [29]. In recent years, a number of clinical studies—focusing on osteoblast metabolism, osteoclast metabolism, and trabecular bone formation in mice—have further confirmed the close association between AMH and bone health. These studies have broken the traditional perception that “AMH only regulates the reproductive system” and verified that AMH can act as a key molecule linking reproductive function, the aging process, and bone metabolism. This provides an important theoretical basis for early risk stratification, mechanism analysis, and the development of new therapeutic targets for osteoporosis [30,31,32]. To systematically clarify the logical progression of AMH research from reproductive regulation to bone metabolism and comprehensively synthesize the latest advances in AMH-related osteoporosis research, three core objectives are set in this review: (1) elaborate the molecular mechanisms by which AMH regulates bone metabolism; (2) integrate evidence from cell experiments, animal models, and clinical studies to verify AMH’s clinical potential as an early screening biomarker and targeted therapeutic target for osteoporosis; (3) analyze current research limitations and propose actionable future directions.

## 2. Biosynthesis, Tissue Expression, Physiological Roles, and Signaling Pathways of AMH

AMH belongs to the transforming growth factor-β (TGF-β) superfamily, and its core function is to induce the regression of Müllerian ducts. Similarly to other TGF-β family proteins, AMH is synthesized as a precursor molecule, and its N-terminal prodomain mediates the folding and dimerization of the C-terminal mature domain [33,34,35]. After cleavage by proprotein convertases, the dimeric precursor is secreted from cells in a form non-covalently bound to the prodomain [36].

**Figure 1:**
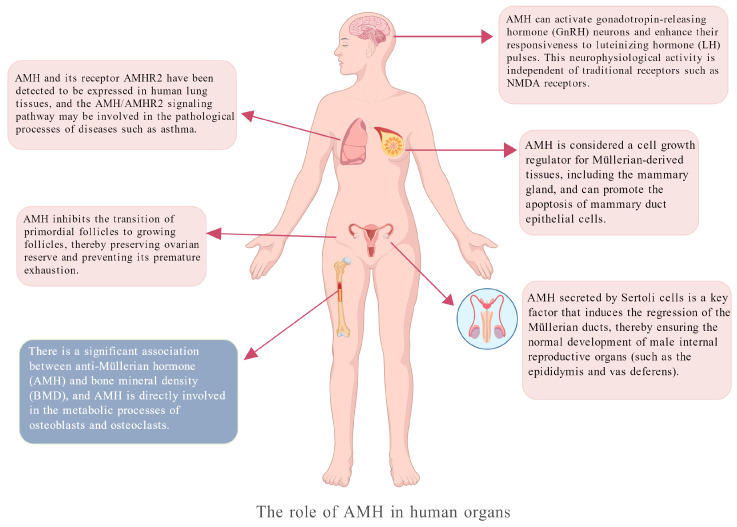
The role of AMH in human organs. AMH is expressed in tissues throughout the human reproductive system, skeletal system, and even the lungs. Its signaling pathways may be implicated in various diseases.

After birth, AMH is secreted in females by the granulosa cells of primary follicles, secondary follicles, and small antral follicles in the ovaries [37]. Its main function is to negatively regulate ovarian function, including inhibiting the initial recruitment of primordial follicles to prevent premature exhaustion of the follicle pool, and reducing the sensitivity of growing follicles to follicle-stimulating hormone (FSH), thereby regulating the selection of dominant follicles [38,39]. Serum AMH levels in women reach a peak at approximately 25 years of age, then decrease steadily, and become undetectable at menopause [40]. Therefore, serum AMH has become a reliable clinical indicator for evaluating female ovarian function (including the quantity and quality of primordial follicles), predicting fertility potential, and assessing the outcome of Assisted Reproductive Technology (ART) [41,42]. Women with polycystic ovary syndrome (PCOS) usually have AMH levels 2–3 times higher than those of normal women (or even higher) due to the presence of a large number of small antral follicles in the ovaries [43,44]. In contrast, women with diminished ovarian reserve (DOR) and premature ovarian insufficiency (POI) have significantly reduced AMH levels [45,46]. During early male embryonic development, AMH is secreted in large quantities by the supporting cells of the fetal testes. Its primary function is to induce the regression and degeneration of the Müllerian ducts (the precursors of the female internal reproductive organs), thereby clearing the path for the Wolffian ducts to develop into the male internal reproductive system (such as the epididymis, vas deferens, and seminal vesicles) [39,47,48]. Additionally, AMH expression has been reported in non-reproductive tissues (e.g., motor neurons, gonadotropin-releasing hormone (GnRH) neurons, or hippocampus), while trace amounts have been detected in the skeletal muscle, sciatic nerve, spinal cord, and mouse brain [49,50,51,52,53]. However, the functional relevance of AMH in these non-reproductive tissues remains incompletely elucidated (Figure 1).

**Figure 2:**
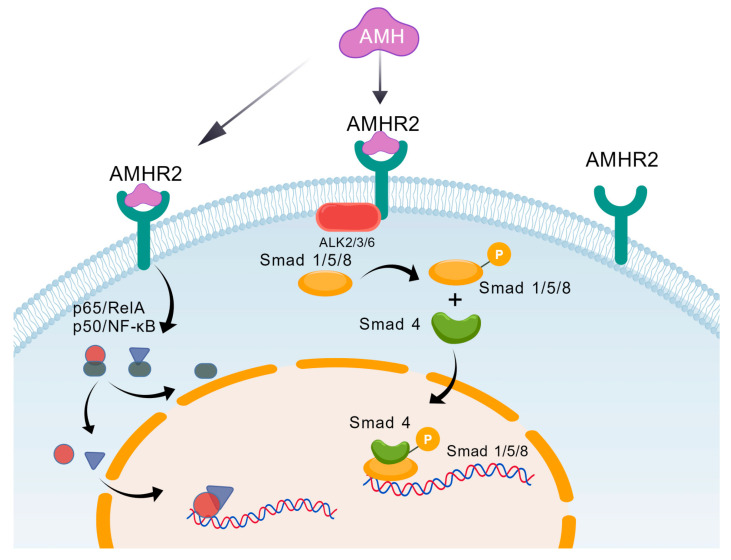
Schematic diagram of the AMH molecular regulatory pathway. AMH first binds to the cell surface AMHR2 receptor and induces its dimerization. It recruits and activates type I receptors such as ALK2/3/6, which subsequently phosphorylate Smad1/5/8. These phosphorylated Smad proteins form a complex with Smad4 and translocate into the nucleus, where they regulate target gene expression to achieve biological effects. The non-Smad pathway of AMH regulates cell proliferation and differentiation by activating the ESR2/p38-MAPK and NF-κB pathways.

The signal transduction of AMH in vivo mainly occurs through two pathways. The classic SMAD-dependent signaling pathway involves AMH binding to the AMH receptor (AMHR2) on the cell surface, thereby activating downstream signaling pathways [54]. AMHR2 is a transmembrane protein belonging to the transforming growth factor-β (TGF-β) receptor superfamily [55]. When AMH binds to AMHR2, the receptor undergoes dimerization, leading to the activation of its intracellular domain’s serine/threonine kinase activity [56]. The AMH-AMHR2 complex subsequently recruits and phosphorylates type I receptors (primarily ALK2, ALK3, or ALK6) [57,58]. Activated type I receptor kinase further phosphorylates intracellular receptor-modulating SMAD proteins (R-SMADs). Phosphorylated R-SMADs form heterodimeric complexes with the common SMAD protein (Co-SMAD) SMAD4 and translocate into the nucleus. There, they function as transcription factors to regulate the expression of a series of target genes, ultimately achieving the biological effects of AMH, refs. [59,60,61,62] as shown in Figure 2. The classic Smad signaling pathway of AMH constitutes a direct signaling cascade from the cell membrane to the nucleus. Its high degree of conservation and precise regulation ensure that AMH can perform its critical physiological functions within specific spatiotemporal contexts. Beyond the Smad pathway, studies have also revealed that AMH can activate other signaling pathways, forming a complex signaling network [63,64]. Following AMH treatment of certain cells (such as ovarian cancer cells), elevated phosphorylation levels of Akt and ERK proteins can be detected, indicating activation of these two pathways [64]. Another study indicated that AMH regulates granulosa cell function through the p38 MAPK signaling pathway [65], and Hsun-Ming Chang et al. discovered that, in breast cancer cells, AMH can activate the NF-κB signaling pathway [66]. These findings suggest that AMH may partially rely on these non-Smad pathways when regulating cell survival and function. Research on the non-Smad signaling mechanisms of AMH remains in its infancy, facing numerous challenges and significant knowledge gaps. However, the Smad and non-Smad signaling pathways of AMH are not two independent parallel lines, but rather form a dynamic, interconnected, and complex signaling network. The interactions between these two pathways, along with their functional division of labor and collaboration, collectively determine the final cellular response to AMH signaling.

## 3. AMH-Mediated Molecular Regulatory Network in Bone Metabolism

### 3.1. The Participation of AMH in the Regulation of Osteoblasts and Osteoclasts

Bone is a highly dynamic tissue that undergoes continuous remodeling [67]. Its renewal and metabolism are precisely regulated through the interaction between osteoblasts (bone-forming cells) and osteoclasts (bone-resorbing cells) [68]. The balance between osteoblast-mediated bone formation and osteoclast-mediated bone resorption is crucial for maintaining stable bone mass. This process involves multiple cytokines and signaling pathways, and previous studies have demonstrated the in vitro regulatory effects of AMH on osteoblasts and osteoclasts [31,32].

Multiple members of the transforming growth factor-β (TGF-β) superfamily play well-defined roles in regulating bone homeostasis [69]. Based on the fact that anti-Müllerian hormone (AMH) is a member of the TGF-β superfamily of glycoproteins, Jung Ha Kim and colleagues investigated whether AMH, like other TGF-β superfamily members, exerts regulatory effects on osteoblasts [31]. The researchers included AMH in a cell culture during osteoblast and osteoclast differentiation processes, observing cellular changes and analyzing the expression levels of osteoblast- or osteoclast-related genes. The results demonstrated that AMH does not affect bone morphogenetic protein 2 (BMP2)-mediated osteoblast differentiation. However, experiments with TRAP staining, mRNA expression levels of osteoblast differentiation markers, and Western blot analysis confirmed that AMH inhibits receptor activator of nuclear factor κB ligand (RANKL)-induced osteoclast differentiation. RANKL and Osteoprotegerin (OPG) form a core molecular pair regulating bone metabolic equilibrium [70]. Together, they constitute the RANK/RANKL/OPG signaling axis, which plays a pivotal role in diverse physiological and pathological processes including bone remodeling, immune regulation, vascular health, and even tumor progression [71]. The RANKL/OPG ratio is a key indicator determining the equilibrium state of bone remodeling [72]. Based on its ability to inhibit osteoclast differentiation, researchers further investigated the effects of AMH on the early signaling pathways of the NF-κB receptor activator ligand (RANKL). Results showed that AMH pretreatment increased the phosphorylation levels of p38 and Erk. Among the multiple signaling pathways activated by RANKL, JNK activation was suppressed, with the most significant inhibition observed in IκB degradation. The above experimental results collectively demonstrate that AMH exerts its inhibitory effect on RANKL-induced osteoclast differentiation by suppressing IκB degradation (while concurrently inhibiting JNK activation). Existing studies have confirmed that Smad1 signaling suppresses osteoclast differentiation: the inhibitory role of TGF-β1 in human osteoclastogenesis is mediated through Smad1 signaling [73]. Moreover, BMP-Smad1 signaling can suppress the initiation of osteoclast differentiation by inhibiting the RANKL-NF-κB pathway [74]. Research by Jung Ha Kim et al. further confirms that AMH negatively regulates osteoclast differentiation by directly inhibiting the RANKL-NF-κB pathway.

In 2023, Francesca Liuzzi et al. conducted in vitro experiments on human osteoblasts (HOb) to evaluate the expression and function of the type 2 anti-Müllerian hormone receptor (AMHRII). They also investigated the effects of exogenous AMH exposure on osteogenic gene expression and osteoblast function [32]. The researchers used granulosa cells as a positive control for AMHRII expression, detecting AMHRII mRNA and protein expression in human osteoblasts (HOb) via RT-PCR and Western blotting, respectively. AMHRII transcripts were detected in both cell models, with no significant difference in mRNA expression levels. Treatment of human osteoblasts with recombinant human AMH (rhAMH) led to elevated pSMAD-1/5 levels following short-term AMH exposure, indicating that AMH mediates its effects in human osteoblasts through BMP-activated SMAD proteins. This study provides the first confirmation of functional AMHRII in human osteoblasts (HOb), offering new insights for future exogenous AMH therapeutic approaches. Furthermore, the research demonstrates that AMH exposure exerts a stimulatory effect on human osteoblasts, activating osteogenic genes (including upregulation of osteogenic transcription factors such as RUNX2 and OSX) while simultaneously increasing mineralization nodule deposition. Thus, novel therapeutic hypotheses can be developed: AMH may serve as an effective candidate factor for treating osteoporosis by specifically targeting osteoblasts and minimizing off-target effects, thereby counteracting bone resorption in patients.

Jung Ha Kim et al. and Francesca Liuzzi et al.’s conflicting findings regarding AMH’s effect on osteoblasts are closely associated with differences in key experimental conditions, which directly influence the detection of AMH’s biological effects (Table 1). Jung Ha Kim et al. used primary osteoblast precursor cells isolated from the cranial vault bones of newborn ICR mice, while Francesca Liuzzi et al. employed the human fetal osteoblast cell line hFOB1.19 (HOb cells).

Species-specific differences in AMH receptor (AMHRII) expression density, signaling pathway sensitivity, and osteogenic differentiation potential may underlie the divergent results. For osteoblast differentiation and mineralization assessment, Jung Ha Kim et al. set short incubation periods (3 days for ALP activity and 6 days for alizarin red staining), while Francesca Liuzzi et al. extended the culture duration to 14 days for mineralized nodule formation. Osteoblast differentiation is a sequential process involving early (ALP activation), middle (osteogenic gene upregulation), and late (mineralization) stages. AMH may exert a delayed or cumulative stimulatory effect on osteoblasts: the 14-day culture in Liuzzi’s study allowed sufficient time for AMH to promote the synthesis and deposition of extracellular matrix, leading to detectable mineralization nodules, whereas the 3–6-day culture in Kim’s study may not have captured this late-stage effect. In terms of gene detection, Liuzzi’s team measured osteogenic gene expression (*RUNX2/OSX*) at frequent intervals (1, 3, 6, and 24 h), enabling the observation of transient peaks in gene expression induced by AMH. In contrast, Kim’s team did not specify frequent gene detection time points, potentially missing the transient upregulation of osteogenic markers and leading to the conclusion that AMH has no effect on osteoblast differentiation. The osteogenic medium used by Francesca Liuzzi et al. contained 10^−7^ M dexamethasone, a classic osteogenic inducer that synergistically promotes osteoblast differentiation by upregulating RUNX2 expression [75,76]. In contrast, Jung Ha Kim et al.’s osteogenic medium lacked dexamethasone, relying only on ascorbic acid and β-glycerophosphate. Dexamethasone may enhance the sensitivity of osteoblasts to AMH: by priming cells for osteogenic differentiation, it creates a favorable microenvironment for AMH to activate downstream Smad signaling, thereby amplifying AMH’s stimulatory effect. Without dexamethasone, mouse primary osteoblast precursors may be less responsive to AMH, resulting in no observable effect on differentiation. Despite these differences in experimental conditions, both studies consistently validate AMH’s core role in bone metabolism: inhibiting osteoclast differentiation and regulating osteoblast function. Their discrepancies highlight the importance of standardized experimental conditions (e.g., unified cell types, consistent culture duration, and standardized medium composition) in future research to ensure the comparability and reproducibility of results. Additionally, complementary studies using primary human osteoblasts, mouse osteoblast cell lines, and in vivo models are needed to comprehensively confirm AMH’s regulatory effects on osteoblasts across different biological contexts.

### 3.2. The Regulatory Role of AMH in the Hypothalamic–Pituitary–Gonadal Axis

The HPG (hypothalamic–pituitary–gonadal) axis is the core regulatory pathway for reproductive function. It begins with gonadotropin-releasing hormone (GnRH) released from the hypothalamus, which acts on the pituitary gland to stimulate the secretion of luteinizing hormone (LH) and follicle-stimulating hormone (FSH), ultimately controlling the production of sex hormones by the gonads [77]. Previous studies have identified the presence of the specific AMH receptor type II (AMHR2) on certain GnRH neurons in the hypothalamus, providing a molecular basis for the central effects of AMH [78]. Functional experiments further confirmed that AMH can directly induce electrical activity in GnRH neurons, increase their firing frequency, and promote pulsatile GnRH release [79]. By modulating GnRH, the terminal signal of the HPG axis, AMH can indirectly influence the secretion patterns of pituitary gonadotropins (LH and FSH) [80]. For example, Ludovica Cotellessa et al. demonstrated in animal studies that AMH enhances GnRH-dependent LH pulsatile secretion [79]. The above studies collectively demonstrate that AMH is a novel upstream regulator of the HPG axis, participating in the precise regulation of reproductive endocrinology through central nervous mechanisms. Since sex hormones (such as LH and FSH) are key regulators of bone metabolism, fluctuations in their levels directly influence the activity of osteoblasts and osteoclasts, ultimately leading to changes in bone mass [81]. Therefore, research on the regulation of the HPG axis by AMH also provides a novel perspective for understanding how AMH influences systemic physiological processes, including bone metabolism. In summary, it can be inferred that AMH directly acts on osteoblasts and osteoclasts within bone tissue to regulate bone remodeling through its specific receptors and downstream signaling pathways (such as inhibiting RANKL and activating Smad). Simultaneously, AMH can also exert effects on the hypothalamus, indirectly influencing skeletal health by modulating systemic hormone levels through its regulation of the entire HPG axis activity. These two pathways may not operate independently but rather synergistically, collectively forming a complex regulatory network. Further exploration of this intricate neuroendocrine–skeletal regulatory network will require additional specific animal studies and clinical intervention trials in the future.

### 3.3. The Role of AMH in the “Reproductive–Bone Axis” Regulatory Network

The “reproductive–skeletal axis” as an emerging endocrinology term has yet to be widely defined, but substantial molecular and clinical evidence indicates the existence of direct communication pathways between the reproductive system (gonads) and the skeletal system that transcend traditional sex hormone-mediated mechanisms [82]. As a hormone primarily secreted by the gonads, AMH is increasingly being revealed as a key messenger molecule linking these two systems. AMH transmits signals by binding to specific receptor complexes on the cell membrane, with its classical signaling pathway similar to other members of the TGF-β superfamily [83,84]. The Smad1/5/8 pathway is commonly regarded as the canonical signaling pathway for bone morphogenetic proteins (BMPs). Furthermore, AMH and BMPs share downstream signaling pathways, providing a crucial molecular biological basis for the ability of AMH to directly influence skeletal development and metabolism [84]. However, this mechanism has not been fully validated in in vivo physiological contexts, and its relative importance compared to other regulatory pathways remains to be clarified.

**Figure 3:**
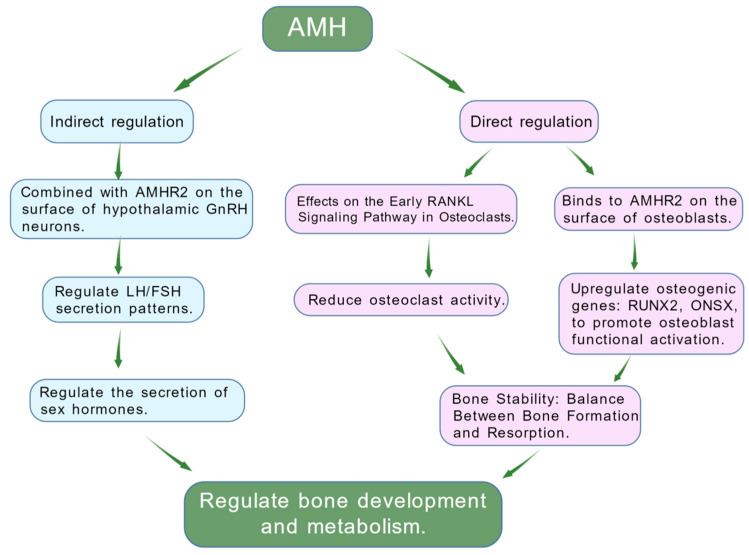
In vivo regulatory network of AMH. AMH regulates bone metabolism through a dual-pathway network. In direct regulation, AMH binds to AMHR2 on osteoblasts to upregulate osteogenic genes, while acting on the RANKL pathway in osteoclasts to reduce their activity. In indirect regulation, AMH binds to receptors on GnRH neurons in the hypothalamus, modulating LH/FSH secretion and sex hormone levels, ultimately synergistically maintaining the balance between bone formation and resorption.

Traditional views hold that the connection between the reproductive system and the skeleton is indirect and primarily mediated through sex hormones (estrogens and androgens) produced by the HPG axis [77]. The concept of the “reproductive–bone axis” proposed in this review aims to describe a direct communication network linking gonadal function status with skeletal metabolic homeostasis. This network is mediated by factors secreted directly by the gonads and operates beyond the traditional effects of sex hormones. Some studies have begun to reveal that bones themselves possess endocrine functions [85]. For example, osteocalcin can negatively regulate testicular function, forming what is termed the “pancreas–bone–testis axis” [86]. These studies further support the notion that complex bidirectional regulatory networks exist between organs (Figure 3). As a hormone secreted directly by the gonads and capable of acting directly on bone, AMH also holds promise as the core molecule for establishing the concept of a “reproductive–bone axis”. However, it is important to acknowledge that AMH is not the sole gonadal factor involved in this potential axis: other gonadal-derived molecules, such as inhibins, activins, and even follicle-stimulating hormone (FSH) (which exerts direct effects on bone cells via its receptors), also contribute to the cross-talk between reproduction and bone metabolism. Future research needs to systematically verify the relative contributions of these factors, clarify their synergistic or independent regulatory mechanisms, and avoid conceptual oversimplification. Only through comprehensive in vivo validation and clinical evidence accumulation can AMH’s exact position in this axis be definitively established.

## 4. Animal Studies on AMH Regulation of Bone Metabolism

In 2022, research published by Christiane van As et al. provided definitive in vivo evidence for AMH’s role in bone metabolism. Utilizing a knockout mouse model deficient in the AMH signaling pathway (AMHKO mice), the researchers systematically evaluated the impact of AMH signaling on bone homeostasis [30]. By utilizing advanced imaging techniques such as micro-computed tomography (Micro-CT), precise quantification of skeletal microstructural parameters was achieved, enabling objective assessment of bone mass changes. The research findings indicate that the absence of AMH signaling leads to gender- and age-dependent changes in bone mass in mice—in particular, a marked loss of trabecular bone. Interestingly, it was also revealed that the skeletal effects of AMH signaling deficiency may be linked to alterations in sex hormone levels (e.g., inhibin B). Its effects thus extend beyond changes in estrogen alone. This suggests that AMH may regulate bone metabolism through pathways independent of or synergistic with traditional sex hormones. This discovery opens a new avenue for research into the mechanisms underlying the role of AMH in bone metabolism.

Although this study is groundbreaking, it also highlights the limitations of current in vivo research, such as evidence primarily relying on single-gene-knockout models and the lack of studies using other types of animal models. At the same time, there is also a relative lack of data from interventional animal studies on whether exogenous AMH supplementation can reverse or improve bone resorption. Therefore, rigorous experimental designs must be employed in future animal research on AMH in bone metabolism to systematically address these unresolved challenges.

## 5. Clinical Correlation Study Between AMH and Osteoporosis

### 5.1. Relationship Between AMH and Bone Mineral Density

Yuzhu Yan et al. investigated the association between serum anti-Müllerian hormone (AMH) and BMD by enrolling 205 premenopausal women aged 30–45 years with suspected ovarian insufficiency [87]. This cross-sectional observational study demonstrated that patients in the low BMD group exhibited significantly lower serum AMH levels compared to the control group (median 0.295 ng/mL vs. 1.730 ng/mL, *p* < 0.05). Correlation analysis revealed a strong negative correlation between serum AMH and age, and a strong positive correlation with BMD/T score. This positive correlation was present in both the low bone density group and the entire cohort, remaining significant after age adjustment (*p* < 0.05). However, no such association was observed in the control group (*p* > 0.05). Multivariate regression analysis revealed that age and AMH are independent predictors of low BMD. Premenopausal women with AMH < 0.800 ng/mL exhibited a 36-fold higher risk of low BMD compared to those with AMH > 0.800 ng/mL. The model achieved 81.0% classification accuracy and 92.0% sensitivity. Serum AMH may thus serve as a potential biomarker for low BMD associated with premenopausal ovarian insufficiency.

A cross-sectional study in India enrolled 300 perimenopausal women aged 40–49 years through community sampling, excluding individuals with diabetes, abnormal liver or kidney function, use of medications affecting bone metabolism, a history of spinal trauma, or polycystic ovary syndrome [88]. Dual-energy X-ray absorptiometry (DXA, Hologic Horizon A, Marlborough, MA, USA) was employed to measure lumbar spine (L1–L4) and femoral neck BMD, vertebral fracture assessment (VFA), and trabecular bone score (TBS) in the enrolled cohort. The results showed that among 300 subjects (mean age 43.2 ± 2.8 years), serum AMH levels were significantly lower in the moderate-to-severe vertebral fracture group compared to the fracture-free group (0.752 ± 0.594 ng/mL vs. 1.023 ± 0.704 ng/mL; *p* = 0.006), while no significant differences were observed in FSH or estradiol; AMH showed significant positive correlations with TBS (r = 0.3; *p* < 0.001), lumbar spine BMD (r = 0.3; *p* < 0.001), and femoral neck BMD (r = 0.2; *p* < 0.001). FSH exhibited significant negative correlations with these indicators, while estradiol showed no significant correlations. Research indicates that the prevalence of severe vertebral fractures among perimenopausal women in India is relatively high (18%), with bone microarchitecture deterioration occurring earlier than bone density decline. As an ovarian reserve marker unaffected by menstrual cycles, AMH exhibits certain predictive value for vertebral fracture risk in this population and may thus serve as a promising indicator for early detection, pending further validation in larger and more diverse cohorts.

Siwen Wang and colleagues conducted a longitudinal cohort study examining the association between anti-Müllerian hormone (AMH) levels during the reproductive years (approximately ages 30–40) and BMD and bone metabolism markers in middle age, approximately 14 years later, providing evidence for predicting long-term bone health in women [89]. The researchers categorized AMH levels into three groups, low (<1.0 ng/mL), normal (1.0–3.5 ng/mL), and high (>3.5 ng/mL), corresponding to different ovarian reserve levels. Dual-energy X-ray absorptiometry (DXA) was used to measure BMD at the lumbar spine (L1–L4), total hip, and femoral neck (primary outcome). Multivariate linear/logistic regression models were adjusted for demographic and lifestyle confounders, supplemented by stratified analysis and mediation effect testing. This longitudinal study demonstrates that midlife AMH levels serve as a predictive indicator of long-term bone health in women. Low AMH levels suggest a higher future risk of bone health issues, providing a basis for the early identification of populations at high risk of osteoporosis and the development of targeted intervention strategies.

The above studies have demonstrated from different regions and different cohort perspectives that AMH levels are closely associated with female BMD, fracture prevalence, and changes in bone microarchitecture. However, the pathological mechanisms linking AMH to low BMD were not explored in these studies, which, moreover, employed observational designs with relatively small sample sizes and did not include endpoint events such as osteoporotic fractures. Future research should validate these findings through longitudinal studies, expanded sample sizes, and multicenter trials, while also delving deeper into the underlying pathological mechanisms to provide more robust evidence for clinical interventions.

### 5.2. AMH as a Predictor of Bone Mass Loss

Arun S. Karlamangla et al. conducted a multicenter, multiethnic prospective cohort study where 888 participants were included in the analysis of future BMD decline rates and 924 in the analysis of sustained bone resorption [15]. The subjects had baseline ages ranging from 42 to 52 years, encompassing premenopausal, early postmenopausal, and late postmenopausal stages. The cohort included non-Hispanic white patients, Black patients, Chinese patients, Japanese patients, and those from other ethnic groups. Multivariate mixed-effects linear regression analysis was used to examine the association between AMH and bone health indicators, stratified by menopausal transition phase (premenopausal, early postmenopausal, and late postmenopausal). The results showed that, in premenopausal and early postmenopausal women, a 50% decrease in AMH levels was associated with a 0.14% acceleration in the annual decline rate of lumbar spine BMD and a 0.11% acceleration in the annual decline rate of femoral neck BMD over the subsequent 3–4 years (both *p* < 0.001). This association was independent of indicators such as FSH and inhibin B. Late perimenopausal AMH levels showed no significant association with future BMD decline rates. ROC analysis indicated that the ability of AMH to identify significant bone resorption (exceeding the minimal clinically significant change value detected by DXA) increases as the menopausal transition progresses. Furthermore, since 81% of women in the late menopausal period exhibit significant bone loss, AMH provides limited additional predictive value for bone loss during this stage. This study demonstrates that AMH levels in premenopausal and early postmenopausal women serve as effective biomarkers for predicting future bone loss, aiding in the early clinical identification of high-risk populations. However, given the baseline age of ≥42 years among participants, the findings do not apply to younger women or those with early menopause. In future research, the population scope should be expanded and follow-up periods extended to further validate the long-term association between AMH and bone health. Additionally, the feasibility of combining AMH with other indicators for predictive purposes should be explored.

### 5.3. Research on the Association Between AMH and Bone Metabolism Biomarkers

Bone turnover markers (BTMs) dynamically reflect the rates of bone formation and resorption [90]. Investigating the relationship between AMH and these markers helps elucidate its specific mechanisms influencing skeletal health. Existing clinical studies have revealed a negative correlation between AMH levels and bone metabolic markers. In a study of premenopausal women with osteoporosis, Yuzhu Yan et al. found that AMH levels showed significant negative correlations with the bone resorption marker type I collagen cross-linked C-terminal peptide (β-CTX), as well as the bone formation markers type I procollagen N-terminal propeptide (TPNPP) and osteocalcin (OC) [87]. This implies that, as AMH levels decline, bone turnover (particularly resorption activity) becomes more active, a typical feature of postmenopausal bone loss. A 2024 study by Siwen Wang et al. further corroborated this finding, showing that lower AMH levels (<1.0 ng/mL) in middle-aged women were significantly associated with elevations in the bone turnover markers PINP and CTX-I [89]. This indicates that a decline in AMH precedes the emergence of clinical symptoms, accompanied by biochemical alterations reflecting a shift in bone metabolism balance toward increased resorption. Therefore, integrating AMH research into clinical settings related to bone metabolism and monitoring its levels may help capture an early “window period” for detecting deteriorating bone health (Table 2).

## 6. Association of AMH with Other Bone-Related Diseases or Metabolites

Recent studies have revealed that beyond osteoporosis, AMH is also closely associated with the development and progression of various other bone-related diseases, as well as the regulation of metabolites. This provides new perspectives for assessing bone health and investigating disease mechanisms (Table 3).

### 6.1. AMH and Idiopathic Bone Marrow Failure Syndrome (IBMFS)

A team affiliated with the National Institutes of Health (NIH) designed a cross-sectional observational study focusing on female patients with Dyskeratosis Congenita (DC) and Diamond–Blackfan Anemia (DBA) within the context of Inherited Bone Marrow Failure Syndromes (IBMFS) [93]. The study population comprised 59 female subjects under 41 years of age, divided into five groups: 15 DC patients, 19 unaffected relatives of DC patients, 12 DBA patients, 13 unaffected relatives of DBA patients, and 16 unrelated healthy female volunteers (serving as controls for both the DC and DBA groups). It was found that female patients with DC and DBA commonly exhibit reduced AMH levels, suggesting diminished ovarian reserve that may increase the risk of infertility and POI (premature ovarian insufficiency). Furthermore, decreased AMH appears to be a shared characteristic among IBMFS conditions (with FA, DC, and DBA all presenting lower AMH levels).

### 6.2. AMH and Inflammatory Bone Diseases

In a study of 518 subjects with early knee osteoarthritis (OA), Eiji Sasaki et al. found that both the early knee OA group and the radiographic knee OA group exhibited significantly lower levels of AMH and estradiol compared to the non-OA group (*p* < 0.05) [92]. Furthermore, AMH showed a positive correlation with BMD (r = 0.491; *p* < 0.05), a negative correlation with bone resorption markers (such as tartrate-resistant acid phosphatase 5b), and a negative correlation with inflammatory markers (such as hyaluronic acid; r = −0.443; *p* < 0.05). This study further demonstrated an association between decreased serum AMH levels during the menopausal transition in middle-aged women and early-stage knee osteoarthritis (OA). Consequently, AMH may serve as a potential biomarker for early-stage knee OA in this population, and its independence from menstrual cycle fluctuations enhances its utility for clinical monitoring.

Rheumatoid arthritis (RA) is an autoimmune disease characterized by synovitis and joint bone destruction. It has been found that serum AMH levels in RA patients show a significant negative correlation with disease activity (such as DAS28 scores) and inflammation markers (such as CRP and ESR) [92]. This implies that, as inflammatory responses become more severe and disease activity increases in RA patients, their serum AMH levels conversely decrease. This suggests that systemic inflammatory states may suppress ovarian function, leading to reduced AMH secretion. However, it is important to emphasize that current research has primarily focused on serum AMH levels. Direct immunohistochemical evidence regarding the expression levels of AMH in local bone or synovial tissues of RA patients and its relationship with local bone erosion remains lacking.

### 6.3. The Regulatory Relationship Between Vitamin D and AMH

Vitamin D is a fat-soluble steroid hormone that plays a crucial role in maintaining bone health [91]. It is not only the core hormone regulating calcium–phosphorus homeostasis but also directly and indirectly modulates the functions of key cells (osteoblasts and osteoclasts) in bone remodeling through complex molecular networks [94]. Nicola A. Dennis et al. employed a design combining correlational analysis with intervention studies, incorporating three independent cohorts: 113 healthy adult males aged 54–93 years, 33 premenopausal women aged 19–39 years, and 74 boys aged 5–6 years [95]. The results showed that serum AMH levels in adult males were significantly positively correlated with 25(OH)D (r = 0.22; *p* = 0.02). AMH and 25(OH)D levels in females exhibited seasonal fluctuations. Serum 25(OH)D levels in 5- to 6-year-old boys showed a wide range of variation (3–237 nmol/L), but no significant correlation was observed between AMH and 25(OH)D (r = 0.07; *p* = 0.54). This indicates that vitamin D may serve as a positive regulator of anti-Müllerian hormone (AMH) levels in adult males and females. Vitamin D deficiency may lead to reduced AMH levels, thereby interfering with clinical assessments such as ovarian reserve evaluation and menopause prediction. Individuals residing in high-latitude regions, those with limited sun exposure, and those with darker skin tones should pay particular attention to how vitamin D status affects AMH test results.

## 7. Conclusions

Based on existing clinical research evidence, anti-Müllerian hormone (AMH) is a sensitive indicator of ovarian aging, whose levels are significantly correlated with women’s bone health status. In the era of precision medicine, AMH shows clear potential in two core areas of osteoporosis management, which are systematically clarified below.

### 7.1. AMH as a Novel Biomarker for Osteoporosis

As a stable and early ovarian reserve indicator, AMH’s changes precede significant estrogen fluctuations, making it superior to traditional markers for early risk stratification. Clinical studies across regions and cohorts consistently demonstrate that low AMH levels are independently associated with low BMD, high bone turnover, accelerated bone loss, and increased vertebral fracture risk [88,89,92]. These findings support AMH as a potential biomarker for early screening of peri- and postmenopausal osteoporosis, addressing the limitations of existing diagnostic tools (e.g., BMD lag and FRAX inaccuracy) and enabling timely identification of high-risk populations.

### 7.2. AMH as a Potential Therapeutic Target for Osteoporosis

As an endogenous hormone, AMH holds therapeutic potential based on its well-validated mechanisms: it directly inhibits RANKL-induced osteoclast differentiation by suppressing the RANKL-NF-κB pathway [31]; in human osteoblasts, it activates osteogenic genes (*RUNX2* and *OSX*) and promotes mineralization through BMP-Smad1/5 signaling [32]. Theoretically, AMH could act as a dual-function therapeutic agent (inhibiting bone resorption and promoting bone formation) with high specificity—targeting only osteoblasts expressing AMHR2 and thus minimizing off-target effects. Although conflicting results on AMH’s osteoblast regulatory effects exist (attributed to experimental design differences), its bone-protective role has been confirmed by animal studies and clinical correlation data [64].

### 7.3. Current Limitations and Future Challenges

Despite significant progress, the field faces key challenges that require targeted attention.

1. Weak causality of observational studies: Most existing clinical evidence relies on cross-sectional or cohort studies, which can only establish associations rather than definitive causal relationships. Confounding factors such as genetic background, dietary calcium/vitamin D intake, physical activity, and comorbidities (e.g., chronic inflammation) may not be fully adjusted for, potentially exaggerating or masking the direct effect of AMH on bone metabolism. For example, the positive correlation between AMH and BMD observed in premenopausal women could be partially mediated by unmeasured genetic factors that simultaneously regulate ovarian reserve and bone homeostasis.
2. Ethnic variability in correlation patterns: Current studies cover diverse populations (Chinese Han, Indian, and American multiethnic), but notable differences in AMH baseline levels, bone mass peak, and menopausal transition characteristics exist across ethnic groups. For instance, Indian perimenopausal women show a higher prevalence of vertebral fractures (18%) [88] compared to other populations, and their AMH-BMD correlation strength (r = 0.3 for lumbar spine BMD) differs slightly from that in Chinese premenopausal women (strong positive correlation) [87]. These variations suggest that AMH’s predictive cutoff values (e.g., 0.800 ng/mL in Chinese women vs. 1.12 ng/mL in Indian women) may not be universally applicable, highlighting the need for ethnic-specific validation studies.
3. Variability due to blood sampling timing and measurement methods: Although AMH exhibits minimal diurnal and menstrual cycle fluctuations, subtle variations may still exist in different menstrual phases (e.g., early follicular vs. luteal phase) or under physiological stress, which could introduce bias in studies with inconsistent sampling protocols. Additionally, AMH measurement methods differ across studies—including ECLIA (Cobas e 601), ELISA (Ansh Labs AL-124), and CLEIA—with variations in reagent specificity and calibration standards leading to inter-study result discrepancies. For example, the same serum sample may yield different AMH concentrations when tested with different ELISA kits, affecting the comparability of correlation coefficients between studies.
4. Need for validation of fracture prediction rather than just BMD changes: Existing studies primarily link AMH to BMD, bone turnover markers, or vertebral fractures, but high-quality evidence validating AMH’s ability to predict major osteoporotic fractures (e.g., hip fractures) is lacking. BMD is only one determinant of fracture risk, and AMH’s association with bone microarchitecture (e.g., trabecular bone score) and fall risk—key fracture predictors—remains understudied.
5. Unclear mechanism of AMH–bone metabolism interaction: While cell and animal studies suggest direct effects of AMH on osteoblasts and osteoclasts, the exact molecular pathways mediating AMH’s role in human bone metabolism are not fully elucidated. Conflicting findings on AMH’s effect on osteoblasts may stem from differences in cell types (human vs. mouse), AMH concentrations, or culture conditions, emphasizing the need for standardized experimental protocols.

### 7.4. Specific Future Research Directions and Clinical Translation Pathways

Targeting the limitations of single-marker prediction, future research should focus on constructing an AMH-based integrated risk assessment model. This model will integrate AMH levels with three types of key indicators: (1) bone microstructural parameters (e.g., trabecular bone score TBS or Micro-CT-derived trabecular thickness); (2) modifiable lifestyle factors (e.g., serum 25(OH)D concentration or weekly weight-bearing exercise duration); (3) genetic susceptibility markers (e.g., ESR1 rs2234693 or LRP5 rs3736228 polymorphisms). After validation in large-scale multiethnic cohorts (including Chinese Han, Indian, and American multiethnic populations), the model can be packaged into a user-friendly web-based or mobile application. This tool will provide personalized osteoporosis risk scores and cutoff values for different ethnic groups, enabling primary care physicians to conduct early screening for premenopausal and perimenopausal women without relying on DXA equipment, thereby improving screening accessibility in resource-limited regions.

As a therapeutic target, AMH-related drugs are expected to undergo phase I/II clinical trials in the future after completing the relevant animal experiments. Based on preclinical evidence of AMH’s dual effects on inhibiting bone resorption and promoting bone formation, the development of two types of therapeutic agents and their clinical trials should be prioritized. (1) Recombinant human AMH (rhAMH) with optimized pharmacokinetics: A randomized, double-blind, placebo-controlled phase I/II trial targeting perimenopausal women with AMH < 1.0 ng/mL and low TBS should be conducted. The trial will evaluate the safety (primary endpoint: incidence of adverse reproductive system events) and efficacy (secondary endpoints: 12-month changes in lumbar spine BMD and bone turnover markers PINP/CTX-I) of subcutaneous injection of rhAMH. (2) Bone-targeted AMHR2 agonists: Liposome or bisphosphonate-modified delivery systems should be utilized to develop AMHR2-selective agonists that accumulate specifically in bone tissue. This approach reduces off-target effects on reproductive organs while enhancing local activation of AMH signaling in osteoblasts/osteoclasts. Preclinical studies will verify its bone-targeting efficiency in ovariectomized mice, followed by phase I trials to assess safety in postmenopausal women with osteoporosis.

To address these challenges, researchers should (1) conduct large-scale, multicenter randomized controlled trials (RCTs) to verify whether AMH-targeted interventions (e.g., recombinant AMH supplementation) can directly improve bone health, establishing causal relationships; (2) perform cross-ethnic studies with standardized sampling timing (e.g., early follicular phase for premenopausal women) and unified AMH measurement methods (e.g., harmonized ELISA kits) to determine ethnic-specific AMH cutoff values for osteoporosis screening; (3) integrate AMH with genetic markers and imaging parameters (e.g., Micro-CT-derived bone microarchitecture) to develop more robust prediction models for osteoporotic fractures; (4) adopt standardized in vitro experimental designs (e.g., consistent cell sources, AMH concentrations, and culture durations) to resolve inconsistencies in AMH’s effect on osteoblasts, complemented by meta-analyses to synthesize existing data.

To resolve conflicting experimental findings, in future molecular studies, researchers should adopt standardized protocols (specifying cell types, AMH concentrations, and culture conditions) and meta-analyses; compare AMH and BMP-2’s signaling activation in human primary bone cells; and use bone cell-specific AMH/AMHR2 knockout animal models to evaluate AMH’s effects under physiological/pathological conditions.

In summary, AMH’s multifaceted relationship with bone metabolism offers new insights into ovarian and systemic aging links. Its dual potential as a biomarker and therapeutic target highlights broad application prospects in women’s lifelong bone health management, but addressing the limitations of observational studies, ethnic variability, and measurement standardization is critical to translating research findings into clinical practice.

## Figures and Tables

**Table 1:** Comparison of experimental designs for AMH regulation of osteoblasts and osteoclasts.

	Francesca Liuzzi et al. [32]	Jung Ha Kim et al. [31]
Experimental results	AMH promotes human osteoblast differentiation and calcification by regulating osteogenic genes.	AMH negatively regulates osteoclast differentiation in mice by inhibiting the RANKL-NF-κB pathway, without affecting osteoblast differentiation.
Cell type	1. Human fetal osteoblast cell line (hFOB1.19, abbreviated as HOb cells) 2. Human granulosa cells (hGCs, AMHRII positive control)	1. Osteoblasts: Primary osteoblast precursor cells from the cranial vault bones of newborn ICR mice 2. Osteoclasts: Bone marrow-derived macrophages (BMMs) from 6-week-old ICR mice
Incubation period	1. Osteogenic gene expression (*RUNX2/OSX*, etc.): 1, 3, 6, 24 h 2. Osteogenic differentiation and mineralization: 14 days 3. Cell viability (proliferation/toxicity): 24, 48, 72 h 4. SMAD activation assay: 30 min–2 h	1. Osteoblast differentiation: 3 days (ALP activity), 6 days (alizarin red staining) 2. Osteoclast differentiation: First cultured with M-CSF for 3 days, then cultured with M-CSF + RANKL for 3 days (total 6 days) 3. Signaling pathway detection: AMH pretreatment for 1 h, followed by RANKL stimulation at 0, 10, and 30 min
Processing factors and concentrations	1. rhAMH: 100 ng/mL 2. Positive control (BMP2): 100 ng/mL 3. Negative control (TGF-β1): 10 ng/mL 4. Osteogenic medium: 50 μg/mL L-ascorbic acid + 10^−7^ M dexamethasone + 10 mM β-glycerophosphate	1. rhAMH: 20/50/100 ng/mL (osteoclasts), 100 ng/mL (osteoblasts) 2. BMP2: 100 ng/mL (osteogenic induction) 3. Osteoclast induction: 30 ng/mL M-CSF + 150 ng/mL RANKL 4. Osteoblast culture medium: 50 μg/mL ascorbic acid + 100 mM β-D-glycerophosphate
Key performance indicators	1. AMHRII: mRNA (RT-PCR), protein (Western blot) 2. Signaling pathways: p-SMAD1/5, p-SMAD3 (Western blot) 3. Osteogenic genes: *RUNX2*, *OSX*, *OPN*, *OC*, *ALP* (real-time PCR) 4. Mineralization: Alizarin red staining (14 days, quantified at 570 nm absorbance) 5. Cell viability: MTT assay, CCK-8 assay	1. Osteogenic-related: ALP activity, Alizarin Red staining, Runx2/Alpl/Ibsp/Bglap (real-time PCR) 2. Osteoclastic-related: TRAP staining (multinucleated cell count), c-fos/Nfatc1/Acp5 (real-time PCR), c-Fos/NFATc1/Cathepsin K (Western blot) 3. Signaling pathways: IκB, p-JNK, p-p38, p-Erk (Western blot) 4. Proliferation: MTT assay
Comparison settings	1. Blank control (no AMH treatment) 2. Positive control (BMP2, activates SMAD1/5) 3. Negative control (TGF-β1, activates SMAD3) 4. hGCs as AMHRII positive control	1. Blank control (no AMH treatment) 2. Osteogenic induction control (BMP2 only) 3. Osteoclastic induction control (M-CSF + RANKL only)

**Table 2:** Clinical studies on the association between AMH and osteoporosis.

	Yuzhu Yan et al. [87] (Premenopausal Chinese Females)	Cijoy Kuriakose et al. [88] (Perimenopausal Female in India)	Siwen Wang et al. [89] (Reproductive-Middle-Aged Women in the United States)	Arun S. Karlamangla et al. [15] (The Menopausal Transition in American Females)
Research Design	Cross-sectional study	Cross-sectional study	Longitudinal cohort study (long-term follow-up)	Longitudinal cohort (repeated observations)
Inclusion population	30–45 years old, premenopausal, 126 individuals (Chinese Han ethnicity)	Perimenopausal women aged 40–49, 300 participants (Indian descent)	From around age 37 during childbearing years to age 51 in middle age, 450 individuals (multiethnic)	Premenopausal/Early Transition Phase (Ages 42–52), 888/924 Participants (Multiethnic)
Tracking time	None	None	Approximately 14 years	3–4 years (bone loss rate)/2–3 years (bone loss volume) after AMH measurement
AMH Testing Method	ECLIA (Cobas e 601)	ELISA	ELISA (Ansh Labs AL-124, Webster, TX, USA)	ELISA (MenoCheck picoAMH ELISA, Ansh Labs, Webster, TX, USA)
Other markers	TP1NP, β-CTX, 25OH-D, PTH	FSH, Estradiol, 25OH-D, PTH, ALP	PINP, CTX-I	FSH, Inhibin B, Urinary NTx
BMD measurement site	Lumbar vertebrae L1–L4	Lumbar spine, femoral neck, forearm	Lumbar spine L1–L4, totality of the hip, femoral neck	Lumbar spine, femoral neck
Additional Bone Assessment	None	Trabecular Bone Score (TBS), Vertebral Fracture (DXA-VFA)	None	Trabecular Bone Score (TBS)
Core Statistical Methods	Spearman correlation, logistic regression, ROC	Spearman correlation, logistic regression, ROC	Linear regression, mediation analysis, stratified analysis (menopause/BMI)	Mixed-effects regression, stratified analysis (menopausal stage), ROC
Association Between AMH and Bone Health	AMH↓→Low BMD risk↑ (OR = 0.2)	AMH↓→Risk of vertebral fracture↑ (OR = 1.85)	AMH↓→Middle-aged BMD↓, BTM↑ (<1.0 vs. >3.5: LS-0.06 g/cm^2^)	AMH↓→Bone loss rate↑ (For every 50% decrease, LS annual decrease +0.14%)
Critical cutoff value	0.800 ng/mL (Sensitivity 78.2%, Specificity 76.9%)	1.12 ng/mL (AUC = 0.8, sensitivity 85%)	1.0 ng/mL (low ovarian reserve threshold)	100 pg/mL (80% sensitivity for early menopause)
Highlights	Focus on Premenopausal Low BMD in a Single-Center Han Chinese Population	Focus on vertebral fractures, combining TBS and VFA	14 years of long-term tracking	Multi-ethnic, postmenopausal women, quantified bone loss rate
Common features	All studies confirm that reduced AMH levels are associated with deteriorating bone health (low BMD, high bone loss, vertebral fractures), and this association remains independent of confounding factors such as age and BMI.			

Note: ↑ denotes increase, ↓ denotes decrease, → denotes causing a certain result to occur.

**Table 3:** Effects of AMH on other bone-related metabolic diseases.

	Nicola A. Denni et al. [91]	Eiji Sasaki et al. [92]	Martha M. Sklavo et al. [93]
Study Population (Gender/Age Range/Sample Size/Basis for Grouping)	Gender: Male, Female, Boys Age: Male 54–93 years, Female 19–39 years, Boys 5–6 years Sample Size: 221 individuals (Male 113 + Female 33 + Male 74) Grouping: Stratified by gender + age (Male/Female/Boy); females additionally stratified by intervention (1000 IU D2/1000 IU D3/Placebo)	Gender: Female Age: 40 years and older Sample size: 518 individuals Grouping: Stratified by “menopausal status (premenopausal/postmenopausal) + OA classification (non-OA/early OA/radiographic OA)”	Gender: Female Age: 41 years and younger Sample size: 65 individuals (DC patients 15 + DC relatives 19 + DBA patients 12 + DBA relatives 13 + healthy volunteers 16) Grouping: Stratified by disease status (DC patients/DBA patients/unaffected relatives/healthy volunteers)
Research Methodology (Research Type/Core Detection Metrics/Statistical Methods)	Study Type: Correlational Study + Interventional Study Core Detection Parameters: Serum AMH (ELISA), Serum 25(OH)D (ELISA for males, LC-MS/MS for females) Statistical Methods: Linear Regression, Partial Correlation Analysis, Paired *t*-Test	Study Type: Cross-sectional observational study Core Assessment Indicators: Serum AMH (CLEIA), sex hormones (CLIA), bone turnover markers, inflammatory markers; knee radiography (KL grading); distal radius BMD (DXA) Statistical Methods: ROC curve analysis, logistic regression, Spearman correlation, ANOVA + Tukey’s post-hoc test	Study Type: Cross-sectional observational study Core Detection Indicator: Serum AMH (Beckman Coulter Gen II ELISA, variability < 10%) Statistical Methods: Mann-Whitney U test (two-group comparison), Kruskal–Wallis test (multiple-group comparison)
Core of the Experiment	Cross-sectional analysis of the correlation between AMH and 25(OH)D in men and boys Six-month supplementation of D2/D3 versus placebo in women during autumn and winter, comparing changes in AMH and 25(OH)D levels between summer and winter and evaluating intervention efficacy	1. Classify 518 women by OA severity and menopausal status, and measure AMH and related biomarkers 2. Determine the cutoff value for AMH in diagnosing early OA using ROC curves 3. Analyze the association between AMH and BMD, bone metabolism/inflammatory markers	1. Select study subjects from the NCI IBMFS cohort, excluding those with ovarian failure or advanced age 2. Measure serum AMH levels and compare differences between patients and controls 3. Compare AMH levels in DC, DBA, and previously diagnosed FA patients (literature data)
Key Findings	1. Males: AMH positively correlates with 25(OH)D (r = 0.22, *p* = 0.02) 2. Females: AMH decreases by 18% in winter compared to summer (*p* = 0.01); D3 blocks this change while D2 is ineffective; AMH changes positively correlated with 25(OH)D changes (r = 0.36, *p* = 0.004) 3. Boys: AMH showed no association with 25(OH)D (r = 0.07, *p* = 0.54)	1. Postmenopausal imaging OA prevalence (48.1%) > premenopausal (12.0%) (*p* < 0.001) 2. AMH cutoff for diagnosing early OA = 0.08 ng/mL (AUC = 0.712, *p* = 0.025); AMH < 0.08 ng/mL indicates high OA risk (OR = 5.55) 3. AMH positively correlates with BMD and negatively correlates with bone metabolism/inflammatory markers	1. DC patients had lower AMH levels (0.55 ng/mL) than relatives (2.28 ng/mL, *p* = 0.004) and healthy individuals (2.69 ng/mL, *p* = 0.005). 2. DBA patients’ AMH (0.89 ng/mL) was lower than controls but not statistically significant 3. FA patients had the lowest AMH (0.05 ng/mL), <DC (*p* = 0.013) and DBA (*p* = 0.003)
Main Findings	Vitamin D positively regulated AMH production in adults. Vitamin D deficiency may interfere with AMH-related clinical diagnoses. Vitamin D status should be considered when obtaining AMH.	Reduced serum AMH levels correlate with early-stage knee osteoarthritis during the menopausal transition in middle-aged women. AMH (cutoff 0.08 ng/mL) may serve as a potential biomarker for early-stage knee osteoarthritis.	Serum AMH levels in women with IBMFS (particularly those with DC) are significantly lower than in healthy individuals. Reduced AMH may represent a common feature of IBMFS or be associated with impaired ovarian reserve and fertility.

## Data Availability

No new data were created or analyzed in this study.
